# Mapping patterns of para-aortic lymph node recurrence in cervical cancer: a retrospective cohort analysis

**DOI:** 10.1186/s13014-021-01856-9

**Published:** 2021-07-10

**Authors:** Bong Kyung Bae, Shin-Hyung Park, Shin Young Jeong, Gun Oh Chong, Mi Young Kim, Jae-Chul Kim

**Affiliations:** 1 Department of Radiation Oncology, School of Medicine, Kyungpook National University, 130 Dongduk-Ro, Jung-Gu, Daegu, 41944 Republic of Korea; 2 Cardiovascular Research Institute, School of Medicine, Kyungpook National University, Daegu, Republic of Korea; 3 Department of Nuclear Medicine, School of Medicine, Kyungpook National University, Daegu, Republic of Korea; 4 Department of Obstetrics and Gynecology, School of Medicine, Kyungpook National University, Daegu, Republic of Korea; 5 Department of Obstetrics and Gynecology, Kyungpook National University Chilgok Hospital, Daegu, Republic of Korea; 6 Clinical Omics Research Center, School of Medicine, Kyungpook National University, Daegu, Republic of Korea; 7 Department of Radiation Oncology, Kyungpook National University Chilgok Hospital, Daegu, Republic of Korea

**Keywords:** Cervical cancer, Recurrent, Para-aortic, Lymph nodes, Clinical target volume

## Abstract

**Background:**

To map anatomic patterns of para-aortic lymph node (PALN) recurrence in cervical cancer patients and validate currently available guidelines on PA clinical target volumes (CTV).

**Methods:**

Cervical cancer patients who developed PALN recurrence were included. The PALNs were classified as left-lateral para-aortic (LPA), aorto-caval (AC), and right para-caval (RPC). Four PA CTVs were contoured for each patient to validate PALN coverage. CTV_RTOG_ was contoured based on the Radiation Therapy Oncology Group guideline. CTV_K_ was contoured as proposed by Keenan et al. CTV_M_ was contoured by expanding symmetrical margins around the aorta and inferior vena cava of 7 mm up to the T12–L1 interspace. CTV_new_ was created by modifying CTV_RTOG_ to obtain better coverage.

**Results:**

We identified 92 PALNs in 35 cervical cancer patients. 46.8% of the PALNs were at LPA, 38.0% were at AC, and 15.2% were at RPC areas. CTV_RTOG_, CTV_K_, and CTV_M_ covered 87.0%, 88.0%, and 62.0% of all PALNs, respectively. PALN recurrence above the left renal vein was associated with PALN involvement at diagnosis (*p* = 0.043). Extending upper border to the superior mesenteric artery allowed the CTV_new_ to cover 96.7% of all PALNs and all nodes in 91.4% of patients.

**Conclusion:**

CTV_RTOG_ and CTV_K_ encompassed most PALN recurrences. For high-risk patients, such as those having PALN involvement at diagnosis, extending the superior border of CTV from the left renal vein to superior mesenteric artery could be considered.

## Background

The current standard of care for locally advanced cervical cancer is external beam radiotherapy and brachytherapy combined with chemotherapy [1]. External beam radiotherapy generally includes the pelvis with or without the para-aortic region. More specifically, nodal clinical target volume (CTV) usually includes external iliac, internal iliac, obturator, and presacral nodal basins. Besides, as the lymphatic system drains from the cervix towards the pelvic and para-aortic nodes, inclusion of the para-aortic (PA) region in the target volume is recommended for patients with common iliac or para-aortic nodal involvement [2, 3].

Although several guidelines for target volume delineation for management of cervical cancer have been reported [4–7], uncertainties in delineating CTV for the PA region still remain. With the advent of modern conformal radiotherapy techniques including intensity-modulated radiotherapy and stereotactic body radiotherapy [8, 9], a clear understanding of target volume definition and consistent contouring that includes PA regions is required. In the past, bony landmarks were used to define the PA region, but those methods might be no longer appropriate in the era of modern radiotherapy.

Previously, studies regarding extended field radiotherapy for cervical cancer only provided partial information in respect to target volume delineation, and PA CTVs varied among those studies [10–13]. Recently, Keenan et al. proposed the first PA CTV contouring guideline, and the authors prospectively validated a proposed PA CTV with a separate patient cohort at the same time [14]. Very recently, the NRG/Radiation Therapy Oncology Group (RTOG) updated consensus guidelines for delineation of CTV for intensity-modulated pelvic radiation therapy in postoperative treatment of endometrial and cervical cancer including the specific PA CTV guideline [15]. However, validations of these currently available guidelines through detailed PA recurrence patterns have been sparse to date.

Herein, we present our work of mapping the anatomic patterns of para-aortic lymph node (PALN) recurrence in cervical cancer patients after definitive therapy in our institution. Additionally, we validate currently available PA CTVs by comparing various CTVs to each enrolled patient in the current study and propose a modification of the CTV.

## Methods

### Patients

Between January 2006 and December 2017, 476 patients with cervical cancer without distant metastasis were treated in our institution. Of those, 82 patients underwent radical hysterectomy and pelvic lymphadenectomy, 171 underwent radical hysterectomy and pelvic lymphadenectomy followed by postoperative radiotherapy, and 223 were treated with definitive radiotherapy. We retrospectively reviewed medical charts of these patients to screen eligible patients for this study. Eligibility criteria included patients treated with either curative-intent surgery or radiotherapy, with or without chemotherapy, and developed PALN recurrence on 2-deoxy-2-[18F] fluorodeoxyglucose (FDG) positron emission tomography/computed tomography (PET/CT) after treatment. A positive finding was defined as a PALN with increased FDG uptake on PET relative to the uptake in comparable normal structures or surrounding tissues, with the exclusion of physiologic bowel and urinary activity. PALNs were considered positive even when the size of the node was smaller than 1 cm if they showed increased FDG uptake. PET/CT scans of patients at the time of PALN recurrence were imported to the treatment planning system for mapping and validating.

Patients who underwent radiotherapy were treated with external beam radiotherapy (EBRT) and/or brachytherapy. EBRT was delivered to the whole pelvis with a four-field box technique or conformal technique using CTV guidelines of pre-existing studies [5, 16]. In patients with PA involvement at initial diagnosis, EBRT included the PA region up to the T12-L1 interspace. For patients with low PA node involvement, adjustments to lower the upper border of extended field radiotherapy were accepted according to the opinion of the attending physician.

### Para-aortic node mapping

Involved PALNs were contoured on individual PET/CT scans. The volumetric center of each lymph node was identified to map anatomic distribution and to validate PA CTV to minimize the size effects of enlarged lymph nodes. All PALNs were classified as left-lateral para-aortic (LPA), aorto-caval (AC), or right para-caval (RPC) considering their relation to the aorta and inferior vena cava (IVC). For classification of PALNs in the vertical direction, the distance from the aortic bifurcation to the left renal vein of an individual patient was measured and divided into thirds and was classified as above left renal vein, upper, middle, or lower third.

A 32-year-old woman with stage IIb cervical cancer was selected as the standard representative patient. The patient’s PET/CT at the time of detection of PALN recurrence served as a template for mapping. The volumetric centers of PALNs of all patients were plotted onto a template using SmartAdapt® deformable image registration algorithm of the Eclipse treatment planning system (Varian Medical Systems, Palo Alto, CA). All mapped PALNs were individually reviewed and adjusted with consideration of distances to anatomic landmarks, including the aorta, IVC, aortic bifurcation, and left renal vein by 2 board-certified radiation oncologists (S.P. and B.B.) and 1 board-certified nuclear medicine physician (S.J.) (with 11 years, 7 years, and 15 years of experience, respectively).

### CTV coverage analysis

**Fig. 1:**
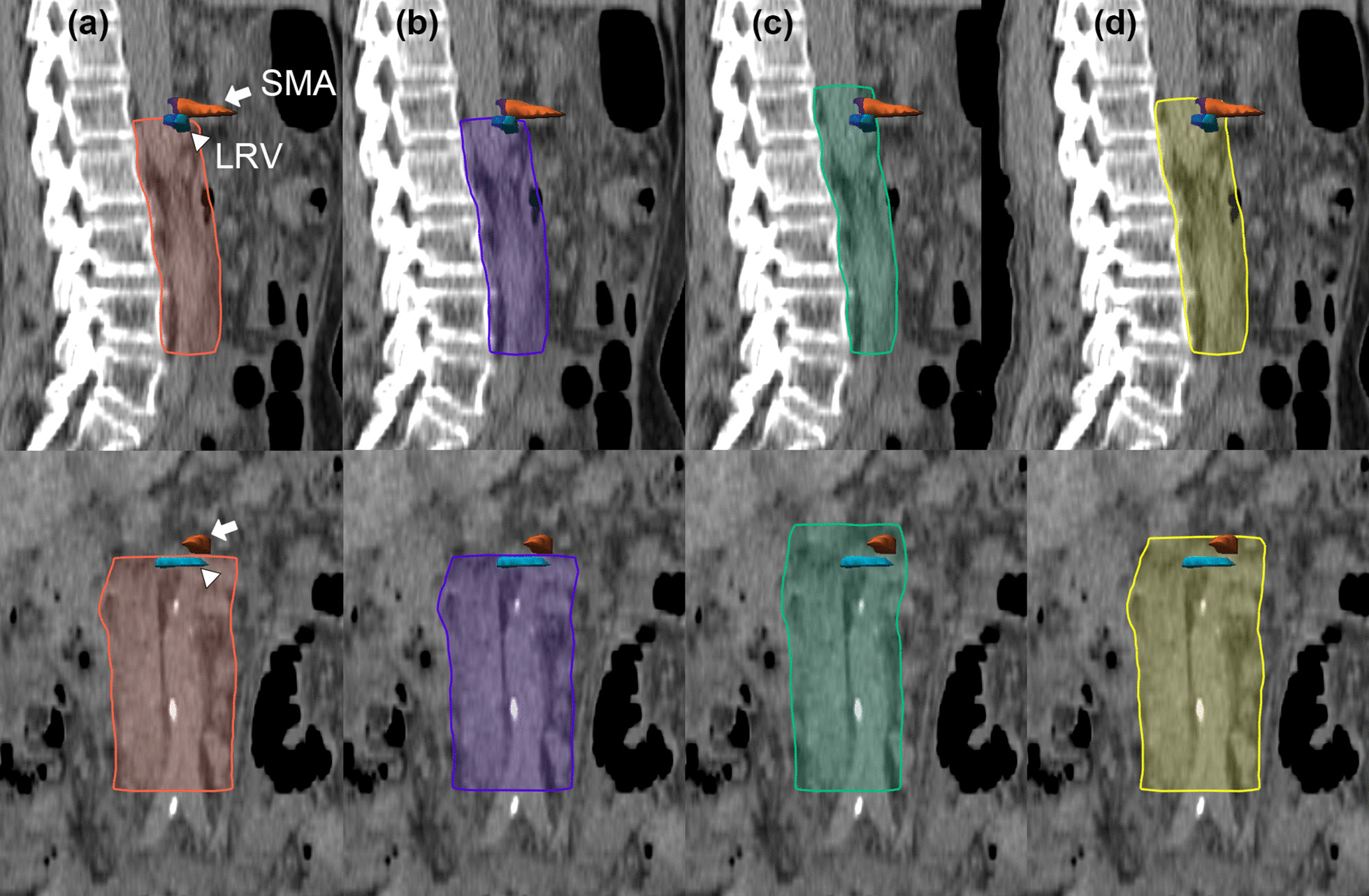
Four clinical target volumes (CTV) for validation analysis of PALN coverage in a representative patient. **a** CTV_RTOG_ (orange), based on the updated NRG/ Radiation Therapy Oncology Group guidelines **b** CTV_K_ (purple), CTV proposed by Keenan et al. **c** CTV_M_ (green), CTV proposed by Milby et al. **d** CTV_new_ (yellow), an extension of CTV_RTOG_ up to the superior mesenteric artery (SMA; arrow). Left renal vein (LRV; arrowhead) is contoured in blue

Three previously proposed CTVs for the PA region were contoured on each patient and were reviewed for validation of PALN coverage. The first PA CTV (CTV_RTOG_) was contoured based on updated RTOG guidelines (expansion from the aorta 10 mm circumferentially, except 15 mm laterally, with further extension to the medial border of the left psoas muscle if needed; expansion from the IVC of 5 mm, up to left renal vein) [15]. The second PA CTV (CTV_K_) was contoured as proposed by Keenan et al. (expansion from the aorta 10 mm circumferentially, except 15 mm laterally; expansion from the IVC of 8 mm anteromedial and 6 mm posterolaterally, up to left renal vein) [14]. The third PA CTV (CTV_M_) was contoured as CTV described by Milby et al. (expanding symmetrical margins around the aorta and IVC of 7 mm, up to T12-L1 interspace) [13]. Finally, we proposed a new CTV (CTV_new_) which was modified based on these validation results to ensure better coverage. Figure 1 shows a representative case with various CTVs contoured for validation of PA CTV coverage.

### Statistical analysis

The chi-square test or Fisher’s exact test was used to compare patients with or without PALN recurrence above the left renal vein. Survival was calculated from the time of recurrence to the date of death or latest follow-up using the Kaplan–Meier method. The log-rank test was used to determine statistically significant factors of survival outcomes. All statistical analyses were performed using R, version 3.2.4 (R Foundation for Statistical Computing, Vienna, Austria).

## Results

### Patients and treatment characteristics

Out of 476 cervical cancer patients who were treated with curative intent, 35 patients with 92 PALN recurrences met the eligibility criteria of the current study. The median age was 47 years (range 24–75 years). The initial stage of patients was Ib in 4 patients, IIa in 1 patient, IIb in 26 patients, IIIb in 3 patients, and IVa in 1 patient. The median primary tumor size was 4.7 cm (range 1.4–11 cm). Initial treatment of patients was definitive surgery alone in 4 patients, definitive CCRT in 25 patients, and surgery with postoperative RT in 6 patients. Among 10 patients who had initial para-aortic node metastases, 5 patients received radiotherapy to PA region up to T12–L1 interspace, 2 patients up to L1–L2 interspace, and 3 patients up to L2–L3 interspace. Median survival time after PALN recurrence was 22.1 months. The median time to PALN recurrence from definitive treatment was 10.0 months (range 2.0–50.9 months). Isolated PALN recurrence was observed in 14 patients (40%), concurrent local recurrence was observed in 8 patients (22.9%), and concurrent distant metastasis was observed in 13 patients (37.1%). No patients were with any recurrences prior to detection of PALN recurrence. The median number of PALN recurrence per patient was 2 (range 1–12). Details of patients and treatments are summarized in Table 1.

**Table 1:** Baseline patient and treatment characteristics

Variables	
Age (median, range)	47 years (24–75)
*Stage*	
I_B_	4 (11.4%)
II_A_	1 (2.9%)
II_B_	26 (74.2%)
III_B_	3 (8.6%)
IV_A_	1 (2.9%)
*Pathology*	
Squamous cell carcinoma	28 (80%)
Adenocarcinoma	2 (5.7%)
Adenosquamous	5 (14.3%)
*HPV infection*	
Positive	16 (45.7%)
Negative	8 (22.9%)
No data	11 (31.4%)
*Primary tumor size (median, range)*	4.7 (1.4–11)
< 4 cm	11 (31.4%)
≥ 4 cm	24 (68.6%)
*Initial lymph node involvement*	
Pelvic	21 (60.0%)
Paraaortic	10 (28.6%)
Negative	8 (22.9%)
*Initial treatment*	
Definitive surgery alone	4 (11.4%)
Definitive CCRT	25 (71.4%)
Surgery + PORT	6 (17.2%)
PALN irradiation for initial treatment	10 (28.6%)
Time to PALN recurrence (median, range)	10.0 months (2.0–50.9)
*Patterns of PALN recurrence*	
Isolated PALN recurrence	14 (40%)
Concurrent local recurrence	8 (22.9%)
Concurrent distant metastasis	13 (37.1%)
No. of PALN per patient (median, range)	2 (1–12)

HPV, human papilloma virus; CCRT, concurrent chemoradiotherapy; PORT, postoperative radiotherapy

### Nodal distribution in respect to anatomic landmarks

**Fig. 2:**
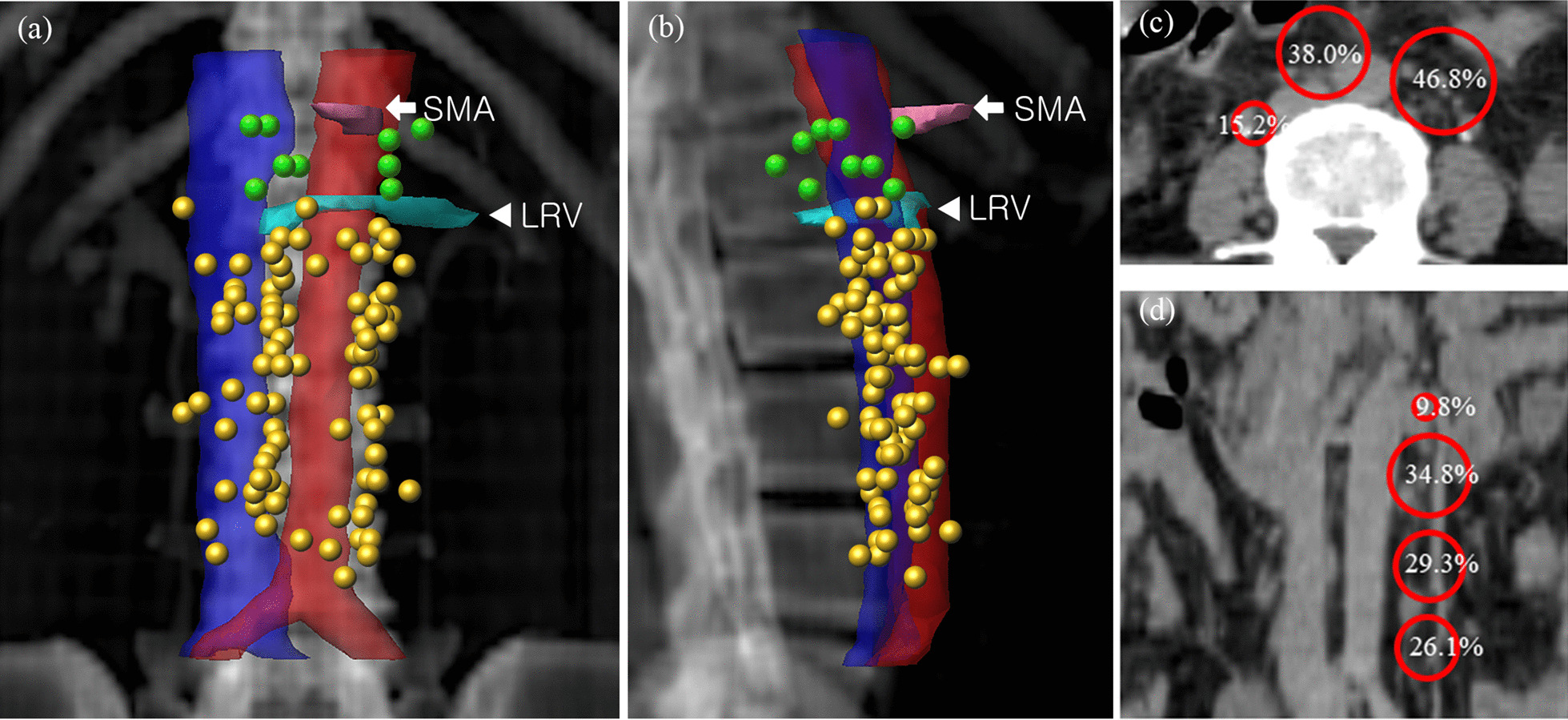
**a**, **b** shows anterior–posterior and lateral views of PALN mapping result onto a representative patient, respectively. PALNs below the left renal vein (LRV; triangle) are plotted in yellow, and PALNs above the left renal vein are plotted in green. **c**, **d** shows the locations of PALNs in horizontal and vertical directions, respectively. Superior mesenteric artery (SMA) is contoured in pink (arrowhead)

A total of 43 PALNs (46.8%) were located on LPA, 35 PALNs (38%) were located on AC, and 14 PALNs (15.2%) were located on RPC. In the vertical direction, 9 PALNs (9.8%) were located above the left renal vein, 32 PALNs (34.8%) were located in the upper third, 27 PALNs (29.3%) were located in the middle third, and 24 PALNs (26.1%) were located in the lower third. The mean ± standard deviation (SD) distance from the aorta to the PALN was 8.2 ± 3.6 mm for LPA, 7.1 ± 3.5 mm for AC, and 21.9 ± 7.5 mm for RPC lymph nodes. The mean ± SD distance from the IVC to the PALN was 27.9 ± 5.5 mm for LPA, 5.8 ± 5.1 mm for AC, and 3.9 ± 2.0 mm for RPC lymph nodes. Details about PALN location and the results of mapping PALN onto the standard representative patient are summarized in Table 2 and Fig. 2.

**Table 2:** Characteristics of recurred PALNs

Variables	
Volume of PALN (mean, SD, range)	4.3 cm^3^ (8.9, 0.2–69.6)
Location of PALN	
*Horizontal*	
LPA	43 (46.8%)
AC	35 (38.0%)
RPC	14 (15.2%)
*Vertical*	
Above left renal vein	9 (9.8%)
Upper	32 (34.8%)
Mid	27 (29.3%)
Lower	24 (26.1%)
Distance of PALNs to major structures (mean, SD, range, mm)	
*LPA*	
Aorta	8.2 (3.6, 2–16.7)
IVC	27.9 (5.5, 15.9–44.2)
*AC*	
Aorta	7.1 (3.5, 1–17.5)
IVC	5.8 (5.1, 1–22)
*RPC*	
Aorta	21.9 (7.5, 10.5–37.3)
IVC	3.9 (2.0, 1.3–9.8)

PALN, para-aortic lymph node; LPA, left para-aortic; AC, aorto-caval; RPC, right para-caval; IVC, inferior vena cava

### Validation of CTV coverage

CTV_RTOG_ covered 80 PALNs (87.0%) and all lymph nodes in 26 patients (74.3%). CTV_K_ covered 81 PALNs (88.0%) and all lymph nodes in 27 patients (77.1%). CTV_M_ covered 57 PALNs (62.0%) and all lymph nodes in 14 patients (40.0%). As illustrated in Fig. 2, showing the volumetric center of the recurrent PALNs in relation to anatomic landmarks, 9 lymph nodes (9.8%) were located above the left renal vein. Most PALNs not covered by CTV_RTOG_ and CTV_K_ (75% and 81.8%, respectively) were located above the left renal vein.

### CTV modification according to the validation results

To cover most of the PALNs based on the validation results, we tried to modify the CTV_RTOG_ by extending the superior border. The new PA CTV (CTV_new_) was contoured by extending the upper borders up to the superior mesenteric artery (SMA) from CTV_RTOG_ (Fig. 1d). CTV_new_ covered 89 PALNs (96.7%) and all lymph nodes in 32 patients (91.4%).

### Factors associated with PALN recurrence above left renal vein

Potential factors for PALN recurrence above left renal vein were investigated (Table 3). Initial PALN involvement was the only factor that was significantly associated with PALN recurrence above left renal vein (*p* = 0.043). Other factors, including tumor size, stage, inclusion of chemotherapy, and initial pelvic lymph node involvement were not associated with PALN recurrence above the left renal vein.

**Table 3:** The comparison results of potential factors related to PALN recurrence above left renal vein

Variables	PALN recurrence above left renal vein		*P* value
	Yes (N = 6)	No (N = 29)	
*Tumor size*			0.664
< 5 cm	3	18	
≥ 5 cm	3	11	
*Number of involved PALN*			0.602
≥ 4	2	6	
< 4	4	23	
*Age*			0.187
≥ 50	1	15	
< 50	5	14	
*Pathology*			1.000
Squamous cell carcinoma	5	23	
Other	1	6	
*Stage*			0.546
III or higher	1	3	
II or lower	5	26	
*HPV*			0.640
Positive	5	19	
Negative	1	10	
*Radiotherapy for initial treatment*			0.128
Included	4	27	
Not included	2	2	
*Chemotherapy for initial treatment*			0.322
Included	3	22	
Not included	3	7	
*Time to PALN recurrence*			1.000
≥ 1 year	2	12	
< 1 year	4	17	
*Concurrent DM*			0.639
Positive	1	10	
Negative	5	19	
*Concurrent LR*			0.602
Positive	2	6	
Negative	4	23	
*Initial pelvic lymph node involvement*			0.191
Positive	2	19	
Negative	4	10	
*Initial PALN involvement*			0.043
Positive	4	6	
Negative	2	23	

PALN, para-aortic lymph node; HPV, human papilloma virus; DM, distant metastasis; LR, local recurrence

## Discussion

Appropriate delineation of the PA region is crucial in radiotherapy for cervical cancer patients, especially in a 3D or IMRT setting, in which a precise target delineation is highly necessary. However, to the best of our knowledge, only a limited number of studies are available about the delineation of the PA region of cervical cancer since the era of conformal radiotherapy [14, 15, 17–19], and no study to date has reported patterns of PA recurrence after curative-intent treatment for cervical cancer. The current study, which presents recurrence patterns of PALNs and validation of PA CTVs proposed in previous studies, could provide further information for appropriate radiotherapy of the PA region in cervical cancer patients.

PALN distribution in relation to major vessels might be the key in appropriate PA CTV delineation. In the horizontal direction, Takiar et al. [18] reported a mean distance from the center of the PALNs to major vessels of 8.3 mm to the aorta and 5.6 mm to the IVC. Keenan et al. [14] reported mean distances from PALNs to the aorta of 8 mm for lymph nodes located at LPA and AC. And mean distances were 6 mm from the IVC to lymph nodes located at AC and 5 mm to lymph nodes located at RPC. In the current study, mean distances from PALNs to the aorta were 8.2 mm for LPA lymph nodes and 7.1 mm for AC lymph nodes, and mean distances from PALNs to the IVC were 5.8 mm for AC lymph nodes and 3.9 mm for RPC lymph nodes. Our results are in agreement with those of Takiar et al. and Keenan et al. [14, 18], showing that mean distances from PALNs to major vessels are different based on the locations of lymph nodes. Therefore, a uniform margin around major vessels, such as the CTV_M_ of our study, seems not suitable for cervical cancer.

Validation results of PA CTVs showed that for horizontal direction, CTV_RTOG_ was the most appropriate CTV for management of PA recurrences in our study. CTV suggested by Keenan et al. (CTV_K_) required an additional margin around the IVC but little benefit compared to CTV_RTOG_ (PALN coverage of 87.0% versus 88.0%). PALN coverage of CTV suggested by Milby et al. (CTV_M_) was unacceptably poor compared to CTV_RTOG_ (PALN coverage of 87.0% versus 62.0%).

The recent update of RTOG guidelines recommended a minimal margin on the right, within 3 to 5 mm around IVC [15]. The reason for recommending a small margin was because there was minimal evidence of nodal involvement to the right of the IVC. However, in the current study, 14 PALNs were located in the RPC region (15.2%). If a 3 mm margin was given around the IVC, only 3 nodes out of 14 (21.4%) were covered by the CTV. Our data suggest at least a 5 mm margin around the IVC is needed to encompass PALNs located on the right of IVC, covering 10 nodes out of 14 (71.4%).

While CTV_RTOG_ generally showed good coverage of recurred lymph nodes, there was a group of patients with recurred lymph nodes above the left renal vein. By extending the upper margin of CTV_RTOG_ from the left renal vein to SMA (CTV_new_), all missed nodes above the left renal vein could be covered. However, routine extension of PA CTV up to SMA could result in excessive toxicity and finding a group of patients who could benefit from extending the upper margin might be required. In the current study, PALN involvement at diagnosis was significantly associated with PALN recurrence above the left renal vein (*p* = 0.043). In general, PALN involvement above the left renal vein at initial diagnosis seems to be rare. Keenan et al. [14] reported that all PALNs were inferior to the left renal vein. Takiar et al. [18] reported that only 4% of PALNs were in the upper third, with 2 lymph nodes located on the T12 level. Kabolizadeh et al. [17] reported that all PALNs were located inferior to or at the level of renal vessels. But the recurrence patterns of the current study show that there are recurred PALNs above the left renal vein, and there may be a group of patients who could benefit from extending the upper border of PA CTV. For patients at high risk of PALN recurrence above the left renal vein, such as patients with PALN involvement at the time of diagnosis, we could carefully consider extending the upper margin of PA CTV up to SMA, instead of the left renal vein.

In the vertical direction, Keenan et al. [14] reported that 2 PALNs (3%) were located on the upper third, 46 (68%) were on the middle third, and 20 (29%) were on the lower third. And the most superiorly located PALN was on the L1 level. Takiar et al. [18] reported that 3 PALNs (4%) were located on the upper third, 26 (36%) were on the middle third, and 43 (60%) were on the lower third. Compared to the vertical location of PALNs in other studies, our data showed a tendency of PALNs to distribute upward (Table 2 and Fig. 2). The difference seems to be related to the patient cohort; while other studies were about cervical cancer patients with PA metastasis at initial diagnosis, our study is about cervical cancer patients with PA recurrence after curative-intent therapy. Curative therapy of cervical cancer includes management of the pelvis, which could lead to irradiating the lower PA region, resulting in a lower incidence of PALN recurrence in the lower third of the PA region in the current study.

There are few potential limitations of the current study. First, due to a small number of patients included, PA CTV validation results could have been biased. Second, other potential risk factors related to PALN recurrence above the left renal vein could have been overestimated or underestimated. And due to the retrospective nature of this study with heterogeneous radiotherapy delivery techniques applied, the results could have been confounded. However, as the current study is about patients with PALN recurrence after definitive treatment for cervical cancer with available PET/CT, we believe 35 patients with 92 PALNs are an acceptable number considering the rigid inclusion criteria. Third, our data needs to be taken with caution because patients with PALN involvement at the time of diagnosis received radiotherapy to PA region. Moreover, due to the retrospective nature of current study, upper border of extended field radiotherapy was not consistent. Nevertheless, it might be meaningful to assess recurrence patterns in patients with initial PA involvement, because it could provide us valuable information how to treat initial PALN-positive patients. Lastly, recurrences were defined based on FDG-avidity on PET/CT, and there could be a potential issue in defining PALN recurrence, about whether an imaging result of PET CT can represent PALN recurrences without pathological confirmation. It is clear that the most exact method to define PALN recurrence is pathologic confirmation by surgical approach. However, a surgical approach provides broad topographic information, and we cannot acquire the information needed for PA CTV contouring such as exact distance from major vessels [20]. That critical information can only be acquired through imaging studies. A meta-analysis by Choi et al. [21] reported that the diagnostic performance of PET/CT was acceptable, with 82% sensitivity and 95% specificity. CT and magnetic resonance imaging showed worse results compared to PET/CT, with 50% sensitivity and 92% specificity, and 56% sensitivity and 91% specificity, respectively. Considering those factors, using PET/CT for detection of PALN recurrence and mapping seems to be a reasonable approach.

In our study, PA CTV based on RTOG guidelines successfully encompassed PALN recurrences in most cases. As distances from major vessels to PALNs were different between locations, a uniform margin around vessels seems not to be appropriate. For high-risk patients, such as having PALN involvement at diagnosis, extending the superior border of the CTV up to the SMA could be considered.

## Data Availability

The data presented in this study are available on request from the corresponding author. The data are not publicly available due to privacy issue.

## Abbreviations

CTV: Clinical target volume
PA: Para-aortic
RTOG: Radiation therapy oncology group
PALN: Para-aortic lymph node
FDG: Fluorodeoxyglucose
PET/CT: Positron emission tomography/computed tomography
EBRT: External beam radiotherapy
LPA: Left-lateral para-aortic
AC: Aorto-caval
RPC: Right para-caval
IVC: Inferior vena cava
SMA: Superior mesenteric artery
